# Ethyl 9-fluoro-5,12-dioxo-5,12-dihydro­indolizino[2,3-*g*]quinoline-6-carboxyl­ate

**DOI:** 10.1107/S160053681202692X

**Published:** 2012-07-25

**Authors:** Da-Li Zhang, Li-Ping Zhang, Jia Yao, Xi-Wei Wu, Lin-Kun An

**Affiliations:** a School of Pharmaceutical Science, Sun Yat-Sen University, Guangzhou 510006, People’s Republic of China; b School of Chemistry and Chemical Engineering, Sun Yat-Sen University, Guangzhou 510275, People’s Republic of China

## Abstract

In the title mol­ecule, C_18_H_11_FN_2_O_4_, the fused four- ring system is essentially planar, with an r.m.s. deviation of 0.032 Å. In the crystal, mol­ecules are connected by π–π stacking inter­actions [centroid–centroid distances = 3.5684 (9) and 3.8247 (9) Å] into chains along [100].

## Related literature

The title compound was obtained in an attempt to synthesize a Top1 (DNA topoisomerase IB) inhibitor. For general background to Top1, see: Pommier (2006 ▶). For the synthesis, see: Shen *et al.* (2008 ▶); Cheng *et al.* (2008 ▶). For a related structure, see: Wu *et al.* (2011 ▶). For the Top1 inhibitory activity of a related indolizinoquinoline-5,12-dione derivative, see: Wu *et al.* (2010 ▶).
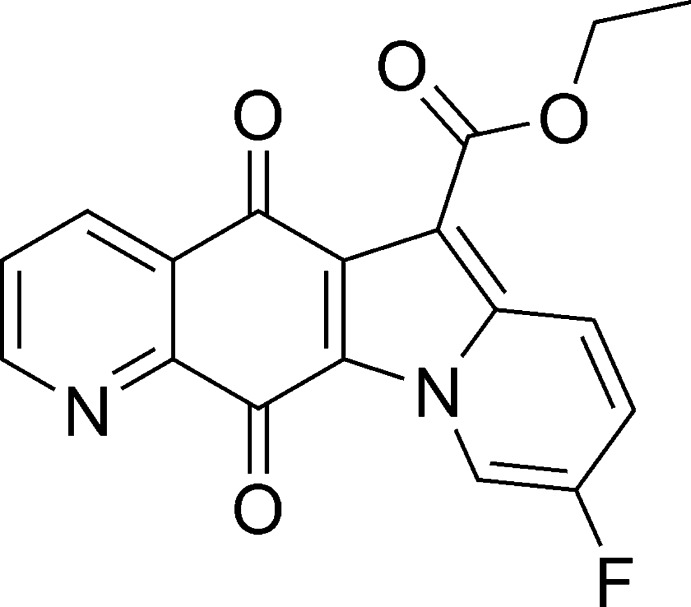


## Experimental

### 

#### Crystal data

C_18_H_11_FN_2_O_4_
*M* *_r_* = 338.29
Monoclinic, 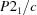

*a* = 6.85562 (10) Å
*b* = 12.12898 (16) Å
*c* = 17.0304 (2) Å
β = 94.2306 (13)°
*V* = 1412.25 (3) Å^3^
*Z* = 4
Cu *K*α radiation
μ = 1.04 mm^−1^
*T* = 136 K
0.30 × 0.20 × 0.20 mm

#### Data collection

Agilent Xcalibur Onyx Nova diffractometer
Absorption correction: multi-scan (*CrysAlis PRO*; Agilent, 2011 ▶) *T*_min_ = 0.550, *T*_max_ = 1.000
5740 measured reflections
2723 independent reflections
2278 reflections with *I* > 2σ(*I*)
*R*_int_ = 0.025

#### Refinement

*R*[*F* ^2^ > 2σ(*F* ^2^)] = 0.037
*wR*(*F* ^2^) = 0.101
*S* = 1.08
2723 reflections
270 parameters
All H-atom parameters refined
Δρ_max_ = 0.30 e Å^−3^
Δρ_min_ = −0.24 e Å^−3^

### 

Data collection: *CrysAlis PRO* (Agilent, 2011 ▶); cell refinement: *CrysAlis PRO*; data reduction: *CrysAlis RED* (Agilent, 2011 ▶); program(s) used to solve structure: *SHELXS97* (Sheldrick, 2008 ▶); program(s) used to refine structure: *SHELXL97* (Sheldrick, 2008 ▶); molecular graphics: *OLEX2* (Dolomanov *et al.*, 2009 ▶); software used to prepare material for publication: *publCIF* (Westrip, 2010 ▶).

## Supplementary Material

Crystal structure: contains datablock(s) I, global. DOI: 10.1107/S160053681202692X/lh5489sup1.cif

Structure factors: contains datablock(s) I. DOI: 10.1107/S160053681202692X/lh5489Isup2.hkl

Supplementary material file. DOI: 10.1107/S160053681202692X/lh5489Isup3.cml

Additional supplementary materials:  crystallographic information; 3D view; checkCIF report

## Figures and Tables

**Fig. 1.**
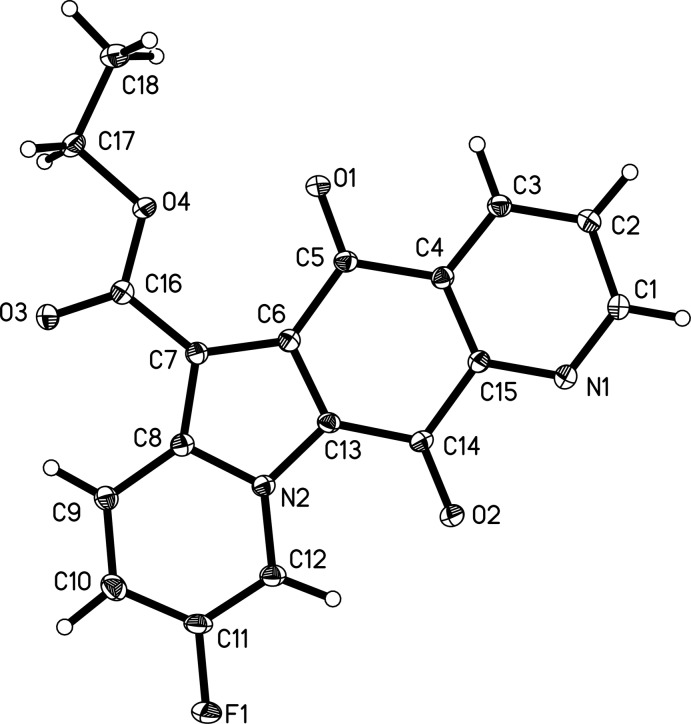
The molecular structure of the title compound. The displacement ellipsoids are at the 30% probability level and H atoms are shown as small spheres of arbitrary radii.

## supplementary crystallographic information

### Comment

Top1 is an essential nuclear enzyme, and can be used as a target to discover anticancer agents (Pommier, 2006). In our previous research, we found ethyl 7-fluoro-5,12-dioxo-5,12-dihydroindolizino[2,3-*g*] quinoline-6-carboxylate is a strong Top1 inhibitor with a different inhibitory mechanism from camptothecin, a well known Top1 inhibitor (Wu *et al.* 2010). In order to investigate the Top1 inhibitory activity of the 9-fluoro substituted isomer, the title compound was synthesized according to a modified literature method (Shen *et al.* 2008; Cheng *et al.* 2008; Wu *et al.* 2011) and its crystal structure was determined.

The asymmetric unit of the title compound is shown in figure 1. In the molecule the four fused aromatic rings system is approximately planar with an r.m.s. deviation = 0.032 Å. In the crystal, molecules are connected by π–π stacking interactions to form chains along [100]. Cg1···Cg1^i^ = 3.5684 (9)Å and Cg1···Cg4^ii^ = 3.8247 (9) Å, where Cg1 and Cg2 are the centroids of the N2/C8/C7/C6/C13 and C4/C5/C6/C13/C14/C15 rings [symmetry codes: (i) -x, 1-y, 1-z, (ii) 1-x, 1-y, 1-z].

### Experimental

According to a modified literature method (Shen *et al.*, 2008; Cheng *et al.*, 2008; Wu *et al.*, 2011), 12 equivalents of 3-fluoropyridine reacted with 6,7-dichloroquinoline-5,8-dione and ethyl acetoacetate to give the title compound as orange solid. Needle-shaped crystals suitable for X-ray analysis were obtained by slow evaporation of a solution of the title compound in chloroform-ethyl acetate (20/1, *v*/*v*).

### Refinement

All H atoms were refined indpendently with isotropic displacement parameters.

### Crystal data

**Table:**

C_18_H_11_FN_2_O_4_	*F*(000) = 696
*M**_r_* = 338.29	*D*_x_ = 1.591 Mg m^−^^3^
Monoclinic, *P*2_1_/*c*	Cu *K*α radiation, λ = 1.5418 Å
Hall symbol: -P 2ybc	Cell parameters from 3543 reflections
*a* = 6.85562 (10) Å	θ = 2.6–72.7°
*b* = 12.12898 (16) Å	µ = 1.04 mm^−^^1^
*c* = 17.0304 (2) Å	*T* = 136 K
β = 94.2306 (13)°	Needle, orange
*V* = 1412.25 (3) Å^3^	0.30 × 0.20 × 0.20 mm
*Z* = 4	

### Data collection

**Table:**

Agilent Xcalibur Onyx Nova diffractometer	2723 independent reflections
Radiation source: Nova (Cu) X-ray Source	2278 reflections with *I* > 2σ(*I*)
Mirror monochromator	*R*_int_ = 0.025
Detector resolution: 8.2417 pixels mm^-1^	θ_max_ = 72.9°, θ_min_ = 5.2°
ω scans	*h* = −8→8
Absorption correction: multi-scan (*CrysAlis PRO*; Agilent, 2011)	*k* = −14→10
*T*_min_ = 0.550, *T*_max_ = 1.000	*l* = −20→20
5740 measured reflections	

### Refinement

**Table:**

Refinement on *F*^2^	Primary atom site location: structure-invariant direct methods
Least-squares matrix: full	Secondary atom site location: difference Fourier map
*R*[*F*^2^ > 2σ(*F*^2^)] = 0.037	Hydrogen site location: inferred from neighbouring sites
*wR*(*F*^2^) = 0.101	All H-atom parameters refined
*S* = 1.08	*w* = 1/[σ^2^(*F*_o_^2^) + (0.0396*P*)^2^ + 0.8533*P*] where *P* = (*F*_o_^2^ + 2*F*_c_^2^)/3
2723 reflections	(Δ/σ)_max_ = 0.001
270 parameters	Δρ_max_ = 0.30 e Å^−^^3^
0 restraints	Δρ_min_ = −0.24 e Å^−^^3^

### Special details

**Table:**

Geometry. All e.s.d.'s (except the e.s.d. in the dihedral angle between two l.s. planes) are estimated using the full covariance matrix. The cell e.s.d.'s are taken into account individually in the estimation of e.s.d.'s in distances, angles and torsion angles; correlations between e.s.d.'s in cell parameters are only used when they are defined by crystal symmetry. An approximate (isotropic) treatment of cell e.s.d.'s is used for estimating e.s.d.'s involving l.s. planes.
Refinement. Refinement of *F*^2^ against ALL reflections. The weighted *R*-factor *wR* and goodness of fit *S* are based on *F*^2^, conventional *R*-factors *R* are based on *F*, with *F* set to zero for negative *F*^2^. The threshold expression of *F*^2^ > σ(*F*^2^) is used only for calculating *R*-factors(gt) *etc*. and is not relevant to the choice of reflections for refinement. *R*-factors based on *F*^2^ are statistically about twice as large as those based on *F*, and *R*- factors based on ALL data will be even larger.

### Fractional atomic coordinates and isotropic or equivalent isotropic displacement parameters (Å^2^)

**Table:**

	*x*	*y*	*z*	*U*_iso_*/*U*_eq_	
F1	0.02823 (18)	0.90196 (9)	0.34624 (7)	0.0382 (3)	
O2	0.28727 (18)	0.79052 (10)	0.60332 (7)	0.0266 (3)	
O3	0.1029 (2)	0.36760 (11)	0.33751 (7)	0.0301 (3)	
O1	0.3911 (2)	0.34756 (10)	0.58977 (7)	0.0307 (3)	
N1	0.4460 (2)	0.68373 (12)	0.73454 (9)	0.0241 (3)	
O4	0.2439 (2)	0.28907 (10)	0.44611 (8)	0.0291 (3)	
C4	0.4124 (2)	0.51043 (14)	0.66509 (10)	0.0199 (4)	
N2	0.1785 (2)	0.66666 (11)	0.45980 (8)	0.0195 (3)	
C13	0.2564 (2)	0.62440 (14)	0.53105 (10)	0.0192 (4)	
C6	0.2735 (2)	0.51065 (14)	0.52314 (10)	0.0193 (4)	
C16	0.1782 (2)	0.37539 (14)	0.40402 (10)	0.0204 (4)	
C14	0.3084 (2)	0.68974 (14)	0.60029 (10)	0.0199 (4)	
C7	0.2028 (2)	0.48181 (14)	0.44537 (10)	0.0199 (4)	
C5	0.3582 (2)	0.44629 (14)	0.59152 (10)	0.0202 (4)	
C15	0.3912 (2)	0.62469 (14)	0.66995 (10)	0.0206 (4)	
C12	0.1396 (3)	0.77509 (15)	0.44053 (11)	0.0238 (4)	
C1	0.5239 (3)	0.62799 (15)	0.79713 (11)	0.0270 (4)	
C10	0.0304 (3)	0.71363 (16)	0.30895 (11)	0.0286 (4)	
C3	0.4925 (2)	0.45453 (15)	0.73164 (10)	0.0223 (4)	
C18	0.2975 (3)	0.09848 (17)	0.46821 (13)	0.0328 (5)	
C8	0.1448 (2)	0.58082 (14)	0.40663 (10)	0.0204 (4)	
C2	0.5502 (3)	0.51463 (15)	0.79815 (11)	0.0253 (4)	
C9	0.0689 (2)	0.60677 (15)	0.32938 (11)	0.0231 (4)	
C17	0.2236 (3)	0.18208 (15)	0.40799 (12)	0.0265 (4)	
C11	0.0667 (3)	0.79580 (15)	0.36591 (11)	0.0266 (4)	
H1	0.568 (3)	0.6713 (18)	0.8436 (12)	0.028 (5)*	
H17A	0.302 (3)	0.1835 (16)	0.3619 (12)	0.023 (5)*	
H2	0.607 (3)	0.4777 (17)	0.8449 (12)	0.026 (5)*	
H9	0.043 (3)	0.5454 (19)	0.2945 (13)	0.033 (6)*	
H3	0.513 (3)	0.3732 (18)	0.7311 (12)	0.031 (5)*	
H12	0.167 (3)	0.8305 (18)	0.4819 (13)	0.032 (6)*	
H10	−0.018 (3)	0.7362 (18)	0.2575 (13)	0.034 (6)*	
H17B	0.079 (3)	0.1699 (18)	0.3898 (12)	0.033 (6)*	
H18A	0.439 (4)	0.117 (2)	0.4875 (15)	0.052 (7)*	
H18B	0.217 (4)	0.099 (2)	0.5139 (15)	0.045 (7)*	
H18C	0.291 (3)	0.022 (2)	0.4457 (13)	0.039 (6)*	

### Atomic displacement parameters (Å^2^)

**Table:**

	*U*^11^	*U*^22^	*U*^33^	*U*^12^	*U*^13^	*U*^23^
F1	0.0529 (7)	0.0221 (6)	0.0372 (7)	0.0044 (5)	−0.0126 (5)	0.0072 (5)
O2	0.0321 (7)	0.0185 (6)	0.0281 (7)	0.0017 (5)	−0.0049 (5)	−0.0011 (5)
O3	0.0403 (7)	0.0246 (7)	0.0237 (7)	−0.0013 (6)	−0.0091 (6)	−0.0024 (5)
O1	0.0468 (8)	0.0173 (7)	0.0265 (7)	0.0026 (6)	−0.0071 (6)	0.0008 (5)
N1	0.0267 (8)	0.0217 (8)	0.0235 (8)	0.0003 (6)	−0.0008 (6)	−0.0025 (6)
O4	0.0408 (8)	0.0168 (6)	0.0278 (7)	0.0042 (6)	−0.0096 (6)	−0.0034 (5)
C4	0.0181 (8)	0.0186 (8)	0.0229 (9)	−0.0005 (6)	0.0003 (6)	−0.0009 (7)
N2	0.0178 (7)	0.0175 (7)	0.0229 (7)	0.0000 (5)	−0.0005 (5)	0.0003 (6)
C13	0.0175 (8)	0.0178 (8)	0.0218 (9)	0.0005 (6)	−0.0015 (6)	0.0025 (6)
C6	0.0169 (8)	0.0202 (8)	0.0208 (8)	0.0004 (6)	0.0009 (6)	−0.0008 (7)
C16	0.0165 (8)	0.0220 (9)	0.0227 (9)	−0.0015 (6)	0.0012 (6)	−0.0008 (7)
C14	0.0183 (8)	0.0160 (8)	0.0252 (9)	−0.0001 (6)	0.0010 (7)	−0.0008 (7)
C7	0.0173 (8)	0.0196 (8)	0.0223 (8)	−0.0005 (6)	−0.0016 (6)	−0.0004 (7)
C5	0.0201 (8)	0.0179 (9)	0.0225 (9)	−0.0007 (7)	0.0012 (7)	0.0019 (7)
C15	0.0212 (8)	0.0181 (8)	0.0219 (9)	−0.0023 (7)	−0.0010 (7)	−0.0002 (7)
C12	0.0235 (9)	0.0192 (9)	0.0284 (9)	0.0015 (7)	−0.0011 (7)	0.0025 (7)
C1	0.0330 (10)	0.0241 (9)	0.0231 (9)	−0.0005 (8)	−0.0046 (8)	−0.0025 (7)
C10	0.0277 (9)	0.0320 (10)	0.0251 (9)	0.0004 (8)	−0.0049 (7)	0.0037 (8)
C3	0.0230 (8)	0.0194 (9)	0.0243 (9)	−0.0006 (7)	0.0003 (7)	0.0020 (7)
C18	0.0386 (11)	0.0218 (10)	0.0375 (12)	0.0030 (8)	−0.0018 (9)	0.0016 (8)
C8	0.0174 (8)	0.0199 (9)	0.0237 (9)	−0.0011 (6)	−0.0001 (7)	−0.0002 (7)
C2	0.0297 (9)	0.0226 (9)	0.0229 (9)	0.0005 (7)	−0.0027 (7)	0.0028 (7)
C9	0.0219 (8)	0.0235 (9)	0.0234 (9)	−0.0002 (7)	−0.0023 (7)	0.0005 (7)
C17	0.0309 (10)	0.0186 (9)	0.0293 (10)	0.0002 (7)	−0.0023 (8)	−0.0053 (7)
C11	0.0286 (9)	0.0183 (9)	0.0320 (10)	0.0021 (7)	−0.0029 (8)	0.0069 (7)

### Geometric parameters (Å, º)

**Table:**

F1—C11	1.352 (2)	C7—C8	1.413 (2)
O2—C14	1.232 (2)	C12—C11	1.354 (3)
O3—C16	1.213 (2)	C12—H12	0.98 (2)
O1—C5	1.219 (2)	C1—C2	1.387 (3)
N1—C15	1.343 (2)	C1—H1	0.98 (2)
N1—C1	1.339 (2)	C10—C9	1.363 (3)
O4—C16	1.329 (2)	C10—C11	1.400 (3)
O4—C17	1.453 (2)	C10—H10	0.95 (2)
C4—C5	1.498 (2)	C3—C2	1.380 (3)
C4—C15	1.397 (2)	C3—H3	1.00 (2)
C4—C3	1.398 (2)	C18—C17	1.503 (3)
N2—C13	1.387 (2)	C18—H18A	1.02 (3)
N2—C12	1.377 (2)	C18—H18B	0.99 (3)
N2—C8	1.388 (2)	C18—H18C	1.00 (2)
C13—C6	1.392 (2)	C8—C9	1.414 (2)
C13—C14	1.443 (2)	C2—H2	0.97 (2)
C6—C7	1.420 (2)	C9—H9	0.96 (2)
C6—C5	1.484 (2)	C17—H17A	0.98 (2)
C16—C7	1.474 (2)	C17—H17B	1.02 (2)
C14—C15	1.501 (2)		
			
C1—N1—C15	116.99 (15)	N1—C1—H1	117.0 (12)
C16—O4—C17	116.44 (13)	C2—C1—H1	119.3 (12)
C15—C4—C5	122.98 (15)	C9—C10—C11	118.60 (17)
C15—C4—C3	118.01 (16)	C9—C10—H10	123.7 (14)
C3—C4—C5	118.99 (15)	C11—C10—H10	117.7 (13)
C13—N2—C8	109.17 (14)	C4—C3—H3	121.3 (12)
C12—N2—C13	128.08 (15)	C2—C3—C4	118.75 (16)
C12—N2—C8	122.75 (15)	C2—C3—H3	119.9 (12)
N2—C13—C6	108.13 (14)	C17—C18—H18A	109.7 (15)
N2—C13—C14	124.58 (15)	C17—C18—H18B	110.7 (14)
C6—C13—C14	127.29 (15)	C17—C18—H18C	111.0 (13)
C13—C6—C7	108.00 (15)	H18A—C18—H18B	109 (2)
C13—C6—C5	118.44 (15)	H18A—C18—H18C	109.6 (19)
C7—C6—C5	133.55 (16)	H18B—C18—H18C	107.2 (19)
O3—C16—O4	123.11 (16)	N2—C8—C7	107.77 (14)
O3—C16—C7	122.68 (16)	N2—C8—C9	118.18 (15)
O4—C16—C7	114.21 (14)	C7—C8—C9	134.04 (16)
O2—C14—C13	123.79 (16)	C1—C2—H2	120.8 (12)
O2—C14—C15	121.81 (15)	C3—C2—C1	118.96 (17)
C13—C14—C15	114.40 (14)	C3—C2—H2	120.2 (12)
C6—C7—C16	132.87 (15)	C10—C9—C8	120.02 (17)
C8—C7—C6	106.93 (14)	C10—C9—H9	123.8 (13)
C8—C7—C16	120.18 (15)	C8—C9—H9	116.1 (13)
O1—C5—C4	119.70 (15)	O4—C17—C18	106.34 (15)
O1—C5—C6	124.04 (16)	O4—C17—H17A	107.5 (12)
C6—C5—C4	116.21 (14)	O4—C17—H17B	108.8 (12)
N1—C15—C4	123.62 (16)	C18—C17—H17A	112.4 (11)
N1—C15—C14	115.71 (15)	C18—C17—H17B	112.2 (12)
C4—C15—C14	120.65 (15)	H17A—C17—H17B	109.3 (16)
N2—C12—H12	117.3 (13)	F1—C11—C12	117.46 (17)
C11—C12—N2	116.90 (17)	F1—C11—C10	118.99 (16)
C11—C12—H12	125.8 (13)	C12—C11—C10	123.55 (17)
N1—C1—C2	123.66 (17)		
			
O2—C14—C15—N1	−2.0 (2)	C14—C13—C6—C5	−1.6 (3)
O2—C14—C15—C4	179.59 (17)	C7—C6—C5—O1	3.6 (3)
O3—C16—C7—C6	175.43 (17)	C7—C6—C5—C4	−179.07 (17)
O3—C16—C7—C8	−2.5 (3)	C7—C8—C9—C10	−178.48 (19)
N1—C1—C2—C3	−0.5 (3)	C5—C4—C15—N1	−177.38 (16)
O4—C16—C7—C6	−4.0 (3)	C5—C4—C15—C14	0.9 (3)
O4—C16—C7—C8	178.07 (15)	C5—C4—C3—C2	177.18 (16)
C4—C3—C2—C1	1.0 (3)	C5—C6—C7—C16	3.4 (3)
N2—C13—C6—C7	−0.53 (19)	C5—C6—C7—C8	−178.46 (17)
N2—C13—C6—C5	178.65 (14)	C15—N1—C1—C2	0.2 (3)
N2—C13—C14—O2	0.6 (3)	C15—C4—C5—O1	175.68 (17)
N2—C13—C14—C15	−179.64 (15)	C15—C4—C5—C6	−1.8 (2)
N2—C12—C11—F1	−179.88 (16)	C15—C4—C3—C2	−1.3 (2)
N2—C12—C11—C10	0.0 (3)	C12—N2—C13—C6	−178.91 (16)
N2—C8—C9—C10	−0.2 (3)	C12—N2—C13—C14	1.3 (3)
C13—N2—C12—C11	178.63 (17)	C12—N2—C8—C7	179.30 (15)
C13—N2—C8—C7	0.03 (18)	C12—N2—C8—C9	0.6 (2)
C13—N2—C8—C9	−178.66 (15)	C1—N1—C15—C4	−0.4 (3)
C13—C6—C7—C16	−177.61 (17)	C1—N1—C15—C14	−178.78 (16)
C13—C6—C7—C8	0.54 (19)	C3—C4—C5—O1	−2.7 (2)
C13—C6—C5—O1	−175.32 (17)	C3—C4—C5—C6	179.87 (15)
C13—C6—C5—C4	2.0 (2)	C3—C4—C15—N1	1.0 (3)
C13—C14—C15—N1	178.18 (15)	C3—C4—C15—C14	179.27 (15)
C13—C14—C15—C4	−0.2 (2)	C8—N2—C13—C6	0.31 (19)
C6—C13—C14—O2	−179.20 (17)	C8—N2—C13—C14	−179.48 (16)
C6—C13—C14—C15	0.6 (3)	C8—N2—C12—C11	−0.5 (3)
C6—C7—C8—N2	−0.35 (18)	C9—C10—C11—F1	−179.75 (17)
C6—C7—C8—C9	178.04 (18)	C9—C10—C11—C12	0.4 (3)
C16—O4—C17—C18	−177.06 (16)	C17—O4—C16—O3	0.8 (3)
C16—C7—C8—N2	178.08 (15)	C17—O4—C16—C7	−179.83 (15)
C16—C7—C8—C9	−3.5 (3)	C11—C10—C9—C8	−0.2 (3)
C14—C13—C6—C7	179.26 (16)		
